# Inversion of Nitrogen Concentration in Apple Canopy Based on UAV Hyperspectral Images

**DOI:** 10.3390/s22093503

**Published:** 2022-05-04

**Authors:** Wei Li, Xicun Zhu, Xinyang Yu, Meixuan Li, Xiaoying Tang, Jie Zhang, Yuliang Xue, Canting Zhang, Yuanmao Jiang

**Affiliations:** 1 College of Resources and Environment, Shandong Agricultural University, Tai’an 271018, China; 2020110359@sdau.edu.cn (W.L.); yuxy.12b@igsnrr.ac.cn (X.Y.); 2019120314@sdau.edu.cn (M.L.); 2020110356@sdau.edu.cn (X.T.); 2020120472@sdau.edu.cn (J.Z.); 2021110200@sdau.edu.cn (Y.X.); 2021110194@sdau.edu.cn (C.Z.); 2 National Engineering Research Center for Efficient Utilization of Soil and Fertilizer Resources, Tai’an 271018, China; 3 National Apple Engineering and Technology Research Center, College of Horticulture Science and Engineering, Shandong Agricultural University, Tai’an 271018, China; ymjiang@sdau.edu.cn

**Keywords:** nitrogen inversion, canopy extraction, UAV, hyperspectral image data, backpropagation neural network, remote sensing

## Abstract

As the major nutrient affecting crop growth, accurate assessing of nitrogen (N) is crucial to precise agricultural management. Although improvements based on ground and satellite data nitrogen in monitoring crops have been made, the application of these technologies is limited by expensive costs, covering small spatial scales and low spatiotemporal resolution. This study strived to explore an effective approach for inversing and mapping the distributions of the canopy nitrogen concentration (CNC) based on Unmanned Aerial Vehicle (UAV) hyperspectral image data in a typical apple orchard area of China. A Cubert UHD185 imaging spectrometer mounted on a UAV was used to obtain the hyperspectral images of the apple canopy. The range of the apple canopy was determined by the threshold method to eliminate the effect of the background spectrum from bare soil and shadow. We analyzed and screened out the spectral parameters sensitive to CNC, including vegetation indices (VIs), random two-band spectral indices, and red-edge parameters. The partial least squares regression (PLSR) and backpropagation neural network (BPNN) were constructed to inverse CNC based on a single spectral parameter or a combination of multiple spectral parameters. The results show that when the thresholds of normalized difference vegetation index (NDVI) and normalized difference canopy shadow index (NDCSI) were set to 0.65 and 0.45, respectively, the canopy’s CNC range could be effectively identified and extracted, which was more refined than random forest classifier (RFC); the correlation between random two-band spectral indices and nitrogen concentration was stronger than that of other spectral parameters; and the BPNN model based on the combination of random two-band spectral indices and red-edge parameters was the optimal model for accurately retrieving CNC. Its modeling determination coefficient (R^2^) and root mean square error (RMSE) were 0.77 and 0.16, respectively; and the validation R^2^ and residual predictive deviation (RPD) were 0.75 and 1.92. The findings of this study can provide a theoretical basis and technical support for the large-scale, rapid, and non-destructive monitoring of apple nutritional status.

## 1. Introduction

Nitrogen is a key element in the growth and development of fruit trees, and it is an important indicator for evaluating the nutritional status of fruit trees [1]. The lack of nitrogen will reduce the concentration of chlorophyll and weaken photosynthesis, which will affect the growth, yield, and quality of fruit trees [2]. How to scientifically monitor fruit trees’ nitrogen status, which clarifies the spatial and temporal distribution, is of great significance to agricultural production [3]. The canopy nitrogen content and nutritional status of apples have traditionally relied on on-ground measurements, which are complex and labor-intensive. Moreover, this method cannot carry out large-scale monitoring. On the other hand, the continuous development of remote sensing (RS) technology provides a fast, non-destructive, and effective method for estimating biophysical and biochemical factors. Therefore, RS-based, non-destructive, and rapid monitoring of nitrogen concentration had become an important topic of current precision agriculture research [4].

Recently, the agriculture application of nitrogen concentration monitoring based on multispectral and hyperspectral RS has considerably increased. However, spectral resolution plays a key role in RS applications [5]. Multispectral RS has discontinuous spectra in the visible and near-infrared bands, which leads to limitations in identifying changes in vegetation spectral characteristics caused by different biochemical parameters or structural characteristics [6]. Hyperspectral RS has a higher spectral resolution than multispectral RS, and it can distinguish the differences in crop spectral characteristics more finely and provides support for the quantitative analysis of physical and chemical parameters such as vegetation nitrogen and chlorophyll [7]. At present, the monitoring of crop physical and chemical parameters based on hyperspectral remote sensing data mainly focused on field crops such as winter wheat, rice, and corn [8,9]. Compared with field crops, the tree is high and discontinuous, and it is more complex in structure, which puzzles the collection and extraction of canopy spectral information. With the development of RS technology, the use of multiple platforms to monitor the biochemical parameters such as chlorophyll and nitrogen concentration of economic forests and fruit trees from the leaf, canopy, and regional levels has also made significant progress [10,11]. Wang et al. [12] used airplanes equipped with HySpex sensors to obtain mixed forest canopy data and evaluated the ability of three VIs to estimate canopy nitrogen concentration (CNC). Poonsak et al. [13] processed the Hyperion data obtained by EO-1 by first-order differentiation and estimated the spatial variability of the nitrogen concentration in the sugarcane canopy. Yang et al. [14] used the FieldSpec^®^ Pro FR spectrometer to collect the hyperspectral data of apple leaves at different growth stages and found that the red region centered at 660 nm and the red border area near 720 nm were more sensitive to changes in nitrogen concentration, and they could monitor changes in crop growth, nutrient stress, chlorophyll, and nitrogen concentration.

However, the platform on which the sensor is mounted limits the application of hyperspectral RS. Ground hyperspectral RS is a discrete point measurement, which cannot achieve continuous observation on a large scale. Hyperspectral satellite remote sensing is easily limited by indicators such as spatial resolution, temporal resolution, and atmospheric conditions, and it is difficult to estimate crop biochemical parameters in a timely and accurate manner [15]. However, UAV platforms have recently been widely used in crop remote sensing monitoring due to their flexibility and convenience [16]. Using UAVs with hyperspectral sensors, remote sensing data with high spatial and spectral resolution can be obtained. These data can effectively ensure the estimation accuracy of biochemical parameters such as nitrogen concentration [17]. It has made UAV platforms increasingly popular in agricultural research and applications. The empirical statistical model, which is based on hyperspectral image data to extract VIs and parameters of spectral characteristic position, is one of the important methods [18,19]. These indices and parameters have a good statistical relationship with some biochemical parameters such as N, and the calculation is simple and easy to obtain [20]. These characteristics make it widely used in the inversion of parameters such as nitrogen concentration [21,22,23]. Researchers [24,25] had tried to use the combination of VIs and feature parameters related to the red edge to invert field crop biochemical parameters. They found that the combination of the two types of spectral parameters significantly improved the accuracy of the inversion model. However, the estimation ability of multi-parameter combination based on hyperspectral image data for apple CNC had yet to be verified. Although hyperspectral images had many advantages, some non-vegetation features in the images affected the expression of spectral information due to factors such as tree planting characteristics and lighting angles.

To address these issues, this study classifies UAV hyperspectral image to effectively extract canopy spectral information, calculates and analyzes spectral parameters, and further optimizes various parameter combinations to explore an effective approach for the inversion and mapping of canopy nitrogen concentration distributions based on hyperspectral image data. An efficient method to identify apple canopy on hyperspectral images is tested, and the spectral reflectance of the apple canopy is extracted. We evaluated the CNC inversion ability of different models based on the VIs, random two-band spectral indices, red-edge parameters, and their combination.

## 2. Materials and Methods

### 2.1. Study Area

**Figure 1:**
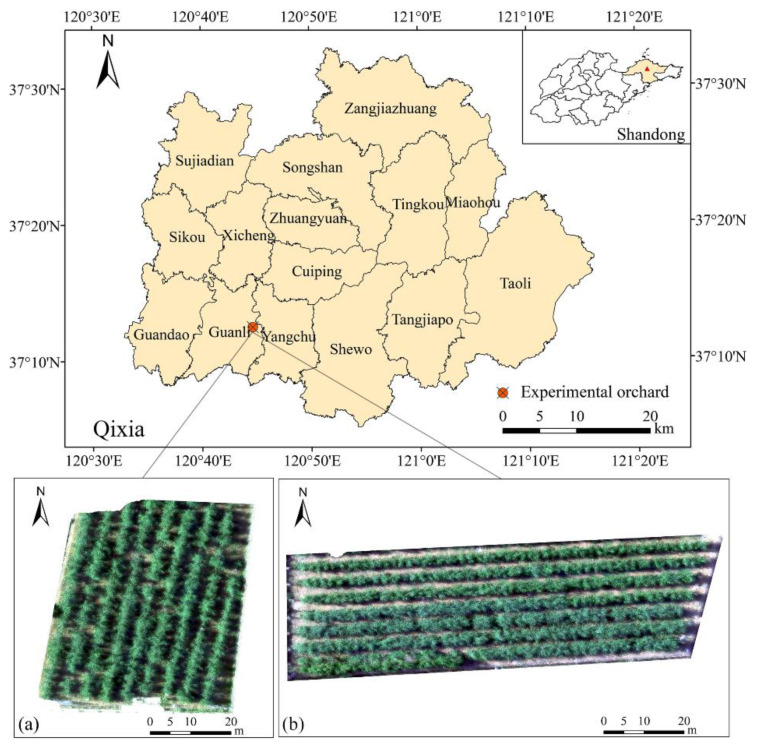
Study area and distribution of sample areas orchards (**a**,**b**). The images of experimental orchards were captured by a UAV and displayed in true color (R_650_, R_562_, R_482_).

Experiments were conducted at apple orchards of the Boshida Group in Guanli town (37°12′50″ N, 120°45′22″ S, Qixia City, Shandong Province in June 2019 (Figure 1). It is located in the middle of the Jiaodong Peninsula and has a warm temperate monsoon climate with an average annual temperature of 11.5 °C. In Qixia, the temperature varies greatly between day and night, the annual sunshine hours are 2660 h, the terrain is dominated by hills, and the soil is mostly brown earth. The dominant apple-producing area in the Bohai Rim, Qixia has concentrated and contiguous apple planting areas, which is ideal for this study. The ‘Red Fuji’ apple tree (Malusdomestica Borkh. cv. ‘Fuji’) was used as the experimental material because it is the main apple cultivar in this region.

### 2.2. Data Acquisition

#### 2.2.1. Field Sampling

Ninety-two apple trees were randomly selected from two ‘Red Fuji’ apple orchards. The growth of these apple trees varied to ensure that the selected samples were representative. We evenly selected 4 vegetative branches around the middle of the apple canopy and collected 3 leaves on each vegetative branch. A total of 12 leaves were collected from each fruit tree, which were healthy and undamaged [15,26]. We took 12 leaves as a whole to represent the nitrogen content of the sample. Moreover, the geographical coordinates of the sampled trees were collected using a Qianxun positioning SR2 satellite-based RTK (Qianxun Spatial Intelligence Inc., Huzhou, China). The sampled leaves were brought back to the laboratory in a cool box. In the laboratory, we first blanched the sample at 105 °C, dried it to constant weight at 80 °C, and then ground it into a powder. Finally, the nitrogen concentration of the leaves was determined by the Kjeldahl method.

#### 2.2.2. Acquisition and Preprocessing of UAV Hyperspectral Image

The UAV platform was a DJI Matrice 600 PRO UAV (loaded mass: 5.5 kg; flying time: 1080 s) (SZ DJI Technology Co., Ltd. Shenzhen, Guangdong Province, China), which was equipped with a Cubert UHD 185-Firefly (UHD185) (Cubert GmbH, Ulm, Baden-Würtemberg, Germany) to collect the hyperspectral image data of apple canopy. This sensor acquired hyperspectral images with wavelengths from 450 to 950 nm and a sampling interval of 4 nm, and it could also obtain a panchromatic image at the same time. To ensure the positional accuracy of the hyperspectral image, nine pieces of 60 cm × 60 cm white reference plates were evenly placed around and in the center of the sample orchard as control points, and their geographical coordinates were recorded for geometric correction. A total of two flights were conducted in 2019 to collect hyperspectral image data under sunny and windless weather. The flight time was 10:00–14:00, and flight missions covering the experimental orchards area were performed with a height of 50 m above the ground, a cruising speed of 5 m/s, a forward overlap of 80%, and a lateral overlap of 60%. Due to the low flying height of the UAV and the uniform and sufficient light during the flight, there was no need for atmospheric correction.

The acquisition procedures of original images are as follows. First, the Cubert Cube-Pilot software version 1.4 (Cubert GmbH) was used to fuse each collected hyperspectral image with the corresponding panchromatic image. Then, the fused single image was stitched by Agisoft PhotoScan (Agisoft LLC, St. Petersburg, Russia). Finally, geometric correction and radiometric calibration were carried out in the processing software ENVI 5.3. We used the reference plate as a control point to correct the geometric distortion of the image and convert the digital number (DN) of the image to the surface reflectance.

#### 2.2.3. Canopy Extraction

When apples were planted, to facilitate field management and receive sufficient light, there are intervals between trees. These factors prevented the apple tree canopy from closing completely. When the sensor and the direct light angle were inconsistent, it also caused shadows to exist on the RS image. The ground between rows (mostly bare soil) and shadows in the image affect the extraction of the information of the canopy, and then, it limits the improvement of the accuracy of the CNC inversion model [27]. Therefore, we needed to distinguish the canopy, bare soil, and shadows clearly in hyperspectral images and extracted only the canopy reflectance to reduce the influence of non-canopy spectral information.

First, the apple canopy hyperspectral image was obtained by setting thresholds for the separately constructed VIs and shadow index to sequentially mask the bare soil and shadows; then, in ENVI5.3, we enter the geographical coordinates of each sample to find the location of its canopy in the hyperspectral image. Finally, the region of interest (ROI) was constructed to extract the reflectance of the canopy of each sample tree, and the average value of all the canopy pixels within a single ROI was taken as the spectral reflectance of a single apple canopy.

### 2.3. Establishment and Verification of CNC Inversion Model

#### 2.3.1. Selection of Spectral Parameters

The spectral response characteristics of vegetation canopy were in the visible, red edge, and near-infrared, and they were closely related to vegetation biochemical parameters. This study selected 16 VIs and red-edge parameters related to vegetation nitrogen to establish the model. The typical VIs included a modified simple ratio (mSR_705_), modified normalized difference (mND_705_), red-edge chlorophyll index (CI_red-edge_), green chlorophyll index (CI_green_), and double-peak canopy nitrogen index (DCNI). The normalized difference spectral index (NDSI), ratio spectral index (RSI), and difference spectral index (DSI) were the main type of random two-band spectral indices. The red-edge position (REP), red-edge amplitude (Dr), minimum red-edge amplitude (Dr_min_), normalized red-edge amplitude (NDDr), ratio of the red-edge amplitude and the minimum red-edge amplitude (RDr), ratio of the red-edge amplitude and the minimum red-edge amplitude (RDr), ratio of the red-edge amplitude and the minimum red-edge amplitude (DDr), and red edge area (SDr) were selected as red-edge parameters in this study. The definition is shown in Table 1.

#### 2.3.2. Analytical Method

We sorted the nitrogen concentration of the samples from small to large and sampled equidistantly at a ratio of 2:1, of which 69 were the modeling set and 23 were the verification set.

This study used two modeling methods: the partial least squares regression (PLSR) and backpropagation neural network (BPNN). PLSR is the result of multiple linear regression, canonical correlation analysis, and principal component analysis [28]. Compared with the traditional regression model, this method uses the sum of squares of minimized errors to construct the optimal model, eliminating the collinearity between variables and improving the accuracy and stability of the model. The BPNN is a feedforward neural network that includes an input layer, an output layer, and multiple hidden layers. Its main feature is that the signal propagates forward and the error propagates back. The returned error is used to continuously adjust the weight of each neuron to obtain the best fit result. BPNN can effectively deal with the nonlinear relationship between data and is widely used in quantitative RS research in the agricultural field [29]. In this work, BPNN has a hidden layer with 4 neurons using the Levenberg–Marquardt (trainlm) algorithm. The activation functions of the hidden layer and output layer are tansig and purelin, respectively. We normalized the data, set the number of iterations to 1000, the learning rate to 0.001, the training target error to 0.0001, and saved the net based on the validation performance. In this work, PLSR and BPNN were performed using the Matlab 2018b software (Math Works, Natick, MA, USA) for estimating the CNC of the apples.

#### 2.3.3. Precision Evaluation

The coefficient of determination (R^2^), root mean square error (RMSE), and relative error (RPD) were used to evaluate the accuracy of the inversion model. Among them, R^2^ can characterize the degree of fit of the model, and RMSE is used to measure the deviation between the predicted value and the measured value. The larger the R^2^, the smaller the RMSE, indicating that the better the prediction effect, the higher the accuracy of the model. RPD is an effective indicator for judging the predictive ability of a model. The prediction performance of this model is poor and cannot be used for prediction analysis when the RPD is less than 1.4. The model has superior predictive power when the RPD is greater than 2.0. The calculation formulas for the values of R^2^, RMSE, and RPD are as follows:

(1) $$R^{2}=\frac{\sum_{i=1}^{N}{\left(\hat{y_{i}}-\bar{\;y\;}\right)}^{2}}{\sqrt{\sum_{i=1}^{N}{\left(y_{i}-\bar{\;y\;}\right)}^{2}}}$$

(2) $$\mathrm{RMSE}=\sqrt{\frac{1}{N}\sum_{i=1}^{N}{\left(y_{i}-\hat{y_{i}}\right)}^{2}}$$

(3) $$\mathrm{SD}=\sqrt{\frac{\sum_{i=1}^{N}{\left(y_{i}-\bar{\;y\;}\right)}^{2}}{N-1}}$$

(4) $$\mathrm{RPD}=\frac{\mathrm{SD}}{\mathrm{RMSE}}$$

where N is the sample size, $y_{i}$ is the measured leaf nitrogen concentration, $\bar{y}$ is the average measured leaf nitrogen concentration, and $\hat{y_{i}}$ is the leaf nitrogen concentration predicted by the model.

## 3. Results

### 3.1. Statistical Results of Nitrogen Concentration

The statistical indicators of nitrogen concentration in the apple canopy, including maximum (Max), minimum (Min), average (Avg), standard deviation (SD), and coefficient of variation (CV), are shown in Table 2. The maximum value of nitrogen concentration in the collected samples was 3.119, the minimum value was 2.121, the average value was 2.622, and the coefficient of variation was 7.360%. These results indicate that nitrogen concentration differs in different apple canopies.

### 3.2. Canopy Extraction and Accuracy Verification

**Figure 2:**
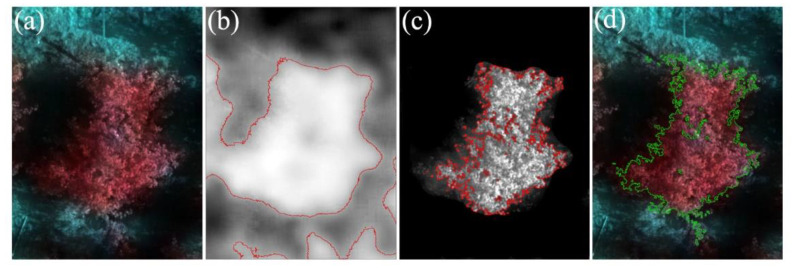
Canopy extraction map of apple: (**a**) Original image standard false color synthesis (R_768_,R_688_,R_628_); (**b**) NDVI 0.65; (**c**) NDCSI 0.45; (**d**) Canopy extraction via RFC.

**Figure 3:**
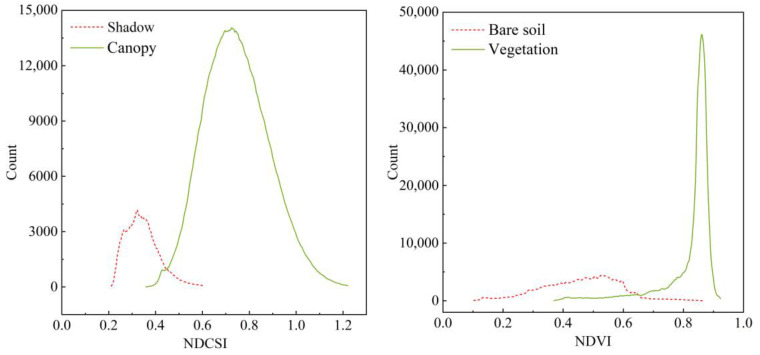
Extraction of VIs threshold. Lines indicate the number of pixels for canopy, bare soil, and shadow in the quadrat, respectively.

The RS image of a single apple tree (Figure 2a) can be divided into three parts: apple canopy, bare soil, and shadow. If we did not distinguish, then the non-canopy features in the ROI would interfere with the extraction of canopy spectrum information. The VIs threshold method is an effective method to distinguish vegetation and non-vegetation pixels [30]. In this study, the vegetation was separated from bare soil by using the most popular NDVI whose band calculation was performed in ENVI to obtain the NDVI map (Figure 2b). However, since NDVI was easy to saturate, the inner shadow of the canopy could not be eliminated. NDCSI [31] used the correlation between the red-edge slope and NDVI to distinguish vegetation and shadows. Histogram thresholding counted the number of pixels at each value of the two ground objects and determined the intersection of the two curves as the threshold according to their distribution. It was a classic method for determining the optimal threshold. As shown in Figure 3, when the NDVI threshold was set to 0.65, the boundary between vegetation and bare soil areas was clear, and the range of the vegetation could be extracted. When the NDCSI threshold was set to 0.45, the effect of removing the inner shadow of the canopy was better, and the purpose of accurately extracting the apple canopy was achieved.

Random forest classifier (RFC) is widely used in RS image classification and information extraction due to its high accuracy, strong stability, and fast calculation speed [32]. We used the RFC tool with the number of trees (N_tree_) parameter set to 500 to classify the hyperspectral image into three categories: canopy, bare soil, and shadow. After processing, the classification result was shown in Figure 2d. Combining the two methods to extract the canopy range for overlay analysis, we found that the threshold method is smaller but more refined than the canopy extracted by RFC. After overlaying the canopy identified by the two methods, we found that the overlap accounts for 96.24% of the threshold method and 86.69% of the RFC. We selected 150 sample points in each of the two orchards to test the accuracy of the two methods. It was verified that in orchard 1, the overall accuracy (OA) and kappa coefficient using the threshold method were 99.65% and 0.9945, respectively. Using RFC, it was 99.44% and 0.9912, respectively. In orchard 2, the OA and kappa coefficient using the threshold method was 99.77% and 0.9952, respectively. Using RFC, it was 99.62% and 0.9923, respectively. The results show that the segmentation accuracy of the threshold method for hyperspectral images is slightly higher than that of RFC. Compared with RFC, the threshold method can improve the classification accuracy by continuously adjusting the threshold size, but its processing process was more complicated. To ensure the accuracy of extracting the average reflectance of the canopy, we finally chose the threshold method to identify the range of the apple canopy in the two orchards.

### 3.3. Relationship between Spectral Parameters and CNC

#### 3.3.1. Correlation Analysis of VIs and CNC

Apple CNC was significantly correlated with spectral parameters (mSR_705_, mND_705_, CI_rededge_, CI_green_, and DCNI), as shown in Table 3. Among them, the correlation coefficients of CI_rededge_ and DCNI were 0.65 and 0.63, respectively, which were significantly higher than other VIs.

Any two bands in the 450–950nm range were combined to construct NDSI, RSI, and DSI, respectively. Then, we performed correlation analysis with CNC for the calculated three parameters. The above calculation was implemented in Matlab 2018b. Random two-band spectral indices with a correlation coefficient greater than 0.45 and the bands that made up it were mostly in the four regions of green, red, red-edge, and near-infrared. The band combined with the largest correlation coefficient was selected for the modeling and verification inversing CNC. The largest correlation coefficient (0.72) was RSI _(466,542)_, which was followed by NDSI _(662,782)_ and DSI _(650,662)_, the correlation coefficients were 0.70 and 0.68, respectively. To screen variables and ensure the accuracy of the model, we selected four spectral indices (RSI, NDSI, DSI, and CI_rededge_) with correlation coefficients greater than or equal to 0.65 to construct a CNC inversion model.

#### 3.3.2. Correlation Analysis of Red-Edge Parameters and CNC

In Table 3, the results showed that the seven red-edge parameters were significantly correlated with the canopy concentration. The highest correlation was NDDr, with a correlation coefficient of 0.69. The correlation level of NDDr and RDr was slightly higher than the typical vegetation indices but lower than random two-band spectral indices. The correlations of other red-edge parameters except for REP are roughly the same as the typical vegetation index. The correlation coefficient between REP and the nitrogen concentration was 0.49, which was the lowest among the selected spectral parameters. However, its reflectivity was the original spectrum. It was reasonable that the correlation coefficient was lower than the processed or calculated spectral parameters. Finally, three red-edge parameters (NDDr, RDr, and SDr) were used as variables to construct the CNC inversion model.

### 3.4. Inversion Model of CNC

#### 3.4.1. Estimation of CNC Based on Single Spectral Parameter

As described above, a unary regression analysis was used for the inversion of CNC for a single spectral parameter. As shown in Table 4, the model based on SDr is linear, while the others are non-linear models. In the modeling dataset, the coefficient of determination of the NDSI-based model was the largest, R^2^ = 0.56, followed by RSI, R^2^ = 0.55, NDDr, and R^2^ = 0.52. The worst model accuracy was the N-CI_rededge_ model, with R^2^ of only 0.35 and RMSE of 0.24. Among the seven models constructed based on a single spectral parameter, although the NDSI-based model modeling set R^2^ was the largest, its verification accuracy is lower, and the RPD was less than 1.4, indicating that the model was not stable and could not estimate the sample. On the contrary, the modeling set and verification set R^2^ of the RSI-based model and the NDDr-based model were relatively similar, indicating that they could estimate samples roughly. In the same way, the remaining four models did not have estimation capabilities, either. The above results show that the model constructed based on a single spectral parameter cannot estimate the CNC.

#### 3.4.2. Estimation of CNC Based on Multiple Spectral Parameters

To test the ability of PLSR and BPNN to estimate CNC, six spectral parameters, NDSI, RSI, DSI, NDDr, RDr, and SDr, were divided into random two-band spectral indices, red-edge parameters, and their combined three categories (Table 5) as variables. Compared with a single spectral parameter, the overall accuracy was significantly higher in the model constructed based on multiple spectral parameters, with R^2^ ranging from 0.54 to 0.77 and RMSE from 0.15 to 0.17. Among them, the BPNN model constructed by using a combination of random two-band spectral indices and red-edge parameters had the best accuracy, with R^2^ values equal to 0.77 and RMSE equal to 0.16. Secondly, the BPNN model based on random two-band spectral indices and the PLSR model based on the vegetation index has R^2^ values of 0.70 and 0.68, respectively. The overall accuracy of the CNC inversion constructed based on BPNN was higher than that of PLSR, indicating that BPNN’s ability to extract information from a variety of spectral parameters and to fit CNC was better than that of PLSR.

**Figure 4:**
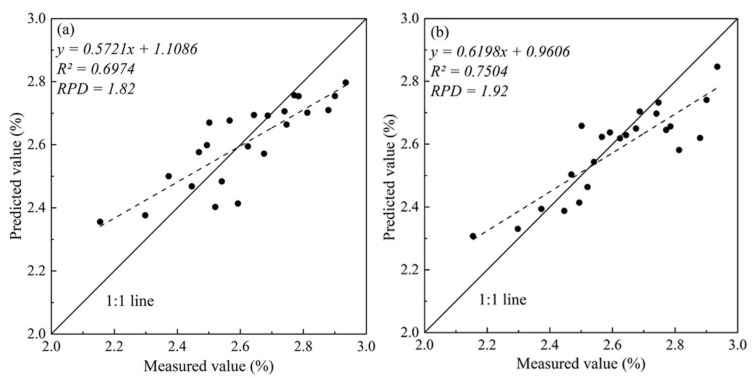
The relationship between CNC measured value and BPNN predicted value based on different variable combinations: (**a**) Random two-band spectral indices; (**b**) Combination of random two-band spectral indices and red-edge parameters.

We used an independent validation dataset (*n* = 23) to verify the reliability of the PLSR and BPNN estimation models. In those models, the BPNN model constructed by using a combination of random two-band spectral indices and red-edge parameters had the best accuracy, RPD = 1.92, and the BPNN model based on random two-band spectral indices is second (RPD was equal to 1.82), indicating that these models have better inversion effects. The RPD of the BPNN model based on the red-edge parameters and the PLSR model based on random two-band spectral indices were 1.63 and 1.58, respectively, indicating that those could estimate samples roughly. The remaining models did not have the ability of inversion. As shown in Figure 4, the closer the drawn point was to the 1:1 line, the better the estimation effect of the model. It could be seen from Figure 4b that the BPNN model constructed by using a combination of random two-band spectral indices and red-edge parameters had a small degree of dispersion. That R^2^ is equal to 0.75, and the slope of the scattered trend line was 0.62. It was the best model constructed in this study. After comparing the accuracy of different spectral parameter combinations and different modeling methods to estimate CNC, we found that the inversion ability based on the combination of multiple spectral parameters was stronger: especially the combination of random two-band spectral indices and red-edge parameters. Moreover, the BPNN that fit the non-linear relationship between multiple spectral parameters and CNC well had adequate inversion accuracy.

### 3.5. Construction of Spatial Distribution Map of CNC

**Figure 5:**
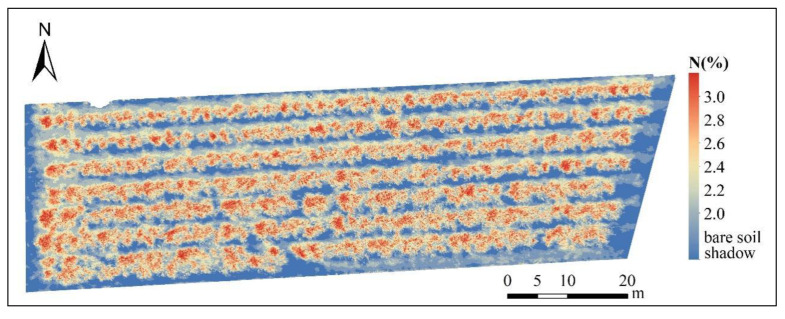
Distribution map of CNC in apple canopy.

As shown in Figure 5, we used the optimal model to perform a spatial inversion on Apple CNC in orchard 2 of the Boshida Plantation Base to verify its feasibility. It can be seen that the CNC in the south of the apple orchard is slightly higher than in the north in terms of spatial distribution. There were differences in the distribution of apple CNC between rows, but the difference in the same row was not obvious. At the level of individual apples, the distribution of CNC was roughly higher at the top and lower at the bottom. The main reason is that different positions receive different light intensities during the growth and development of the canopy The top of the canopy has high light intensity and high chlorophyll concentration, and the photosynthetic rate was more substantial than that at the bottom [33].

## 4. Discussion

As an essential indicator for monitoring vegetation growth, canopy spectrum characteristics can reflect the pigment concentration, nutritional status, and canopy structure of vegetation, providing an important basis for RS to retrieve various biochemical indicators of vegetation [34]. However, in the actual production process, appropriate spacing should be maintained between the rows of apples in the orchard to facilitate management and harvesting. At the same time, this method also improves the light and ventilation conditions of the tree to ensure the growth of apples. Affected by the above factors, the unclosed canopy will make the bare land appear in the image. Furthermore, the three-dimensional structure of the canopy and changes in the direct sun angle also exacerbate this phenomenon. Therefore, the key to extracting canopy spectrum information is accurately classifying RS images to determine the canopy range. We chose the more common and simple vegetation index threshold method to identify and extract the apple canopy. NDVI is currently the most widely used vegetation index, which can be used to extract green vegetation. However, it is easy to saturate, and the effect of removing shadows in the canopy is poor [35]. For this reason, NDCSI was introduced to remove the shadow part of the canopy. We found that when the DNVI and NDCSI thresholds are set to 0.65 and 0.45, respectively, the effect of extracting the canopy range is the best, which is consistent with the results of other studies [36]. We compared the extraction effect of the threshold method and RFC on the canopy. Generally, the extraction range of the two canopies is relatively consistent, and the extraction range of the threshold method is more refined. Since the types of orchard features are relatively simple and the spectral differences of features are more obvious, the optimal range of the canopy can be determined by adjusting the threshold size to extract canopy spectrum information. However, it is more affected by human factors and more complicated; thus, it is suitable for small-scale research compared with RFC.

Using RS data to construct vegetation indices is a simple, commonly used, and effective method to qualitatively and quantitatively evaluate vegetation coverage, growth status, and various biochemical parameters [37]. We selected five vegetation indices and constructed and screened three two-band spectral indices, RSI_(466,542),_ NDSI_(662,782)_, and DSI_(650,662),_ for correlation analysis with the CNC of apples. In this work, the correlation between the vegetation index and nitrogen based on the random two-band combination and the accuracy of the proposed model is higher than the typical vegetation index, but the sensitive wavelength of the composition is different from other studies [15,16]. This is mainly because of the different types of crops, which are quite different in canopy structure and growth period, which causes the shift of the sensitive wavelength of nitrogen absorption. The red edge is the band where the high reflectance from the low reflectance red region of the vegetation spectrum increases sharply to the near-infrared region. REP is the most typical feature of this region, and it is useful for the estimation of nitrogen concentration [38]. Although the estimation ability of CNC based on red-edge parameters was slightly lower than that based on random two-band spectral indices, the combination of those as variables significantly improved the estimation accuracy, which also obtained similar results in the estimation of biochemical parameters of other crops [25]. In this study, there were many bands of hyperspectral RS data, which have rich spectral information, and it can construct an inversion model with high accuracy. We selected five vegetation indices and constructed and screened three two-band spectral indices, RSI_(466,542),_ NDSI_(662,782)_, and DSI_(650,662),_ for correlation analysis with the CNC of apples. In this work, the correlation between the vegetation index and CNC on the random two-band combination and the accuracy of the construction model is higher than the typical vegetation index, but the sensitive wavelength of the composition is different from other studies [15,16]. This is mainly because of the different types of crops, which are quite different in canopy structure and growth period, which causes the shift of the sensitive wavelength of nitrogen absorption. The red edge is the band where the high reflectance from the low reflectance red region of the vegetation spectrum increases sharply to the near-infrared region. REP is the most typical feature of this region, and it is useful for the estimation of nitrogen concentration [38]. Although the estimation ability of CNC based on red-edge parameters was slightly lower than that based on random two-band spectral indices, the combination of those as variables significantly improved the estimation accuracy, which also obtained similar results in the estimation of biochemical parameters [25]. In this study, there are many bands of hyperspectral RS data, which have rich spectral information, and it can construct an inversion model with high accuracy. However, hyperspectral data are redundant and require complex processing, which brings challenges to a larger range of CNC estimation.

## 5. Conclusions

In this work, UAV hyperspectral images were used to build a model of vegetation indices, red-edge parameters, and their combinations to invert and map CNC distributions. It was found that the threshold method based on vegetation indices was an effective method to extract the apple canopy, which can reduce the influence of bare land and shadows on the spectral information of the canopy. The correlation between the spectral index of two random bands and the CNC of apples was better than the red-edge parameters and traditional vegetation indices, and RSI based on the 466 and 542 nm was the most sensitive indicator. Furthermore, it was found that compared with other models, the BPNN model constructed based on the combination of random two-band spectral indices and red-edge parameters could invert apple CNC more accurately. The apple CNC distribution map generated by the optimal model can better reflect the spatial distribution of CNC and provide a theoretical basis and technical support for the monitoring and precise management of the nutrient status of apple trees.

## Figures and Tables

**Table 1:** Spectral parameters and definitions.

Types	Spectral Parameters	Definition
Vegetation indices	mSR_705_	$\left(R_{750}-R_{445}\right)/\left(R_{705}-R_{445}\right)$
	mND_705_	$\left(R_{750}-R_{705}\right)/\left(R_{750}+R_{705}-2R_{445}\right)$
	CI_red-edge_	$\left(R_{840}-R_{870}\right)/\left(R_{720}-R_{730}\right)-1$
	CI_green_	$\left(R_{840}-R_{870}\right)/R_{550}-1$
	DCNI	$\left(R_{722}-R_{702}\right)/\left(R_{702}-R_{670}\right)/\left(R_{722}-R_{670}+0.03\right)$
Random two-band spectral indices	NDSI	$\left(R_{i}-R_{j}\right)/\left(R_{i}+R_{j}\right)$
	RSI	$R_{i}/R_{j}$
	DSI	$R_{i}-R_{j}$
Red-edge parameters	REP	The wavelength of the maximum first derivative of the spectrum in the range of 680–750 nm
	Dr	The first derivative of the red-edge position
	Dr_min_	The wavelength of the minimum first derivative of the spectrum in the range of 680–750 nm
	NDDr	$\left(\mathrm{Dr}-{\mathrm{Dr}}_{\min}\right)/\left(\mathrm{Dr}+{\mathrm{Dr}}_{\min}\right)$
	RDr	$\mathrm{Dr}/{\mathrm{Dr}}_{\min}$
	DDr	$\mathrm{Dr}-{\mathrm{Dr}}_{\min}$
	SDr	The sum of the first derivative of the spectrum of the red-edge region

Note: R is spectral reflectance; D is the first-order differential.

**Table 2:** Statistical indices of nitrogen concentration.

Dataset	Samples	Max/%	Min/%	Avg/%	SD	CV
Total	92	3.119	2.121	2.622	0.193	7.361%
Modeling Set	69	3.119	2.121	2.624	0.194	7.393%
Validation Set	23	2.935	2.155	2.616	0.197	7.531%

Max, Min, Avg, SD, and CV indicate the maximum, minimum, average, standard deviation, and coefficient of variation of the apple fruit yield, respectively.

**Table 3:** Correlation between spectral parameters and CNC.

Types	Spectral Parameters	Sensitive Wavelength (nm)	Correlation
Vegetation indices	mSR_705_	$R_{445},{\;R}_{750}$	0.59 **
	mND_705_	$R_{445},R_{705},R_{750}$	0.52 **
	CI_rededge_	R_720_,R_730_,R_840_,R_870_	0.65 **
	CI_green_	$R_{550},R_{840},R_{870}$	0.60 **
	DCNI	$R_{670},R_{702},R_{722}$	0.63 **
Random two-band spectral indices	NDSI	$R_{662},R_{782}$	0.70 **
	RSI	$R_{466},R_{542}$	0.72 **
	DSI	$R_{650},R_{662}$	0.68 **
Red-edge parameters	REP	$R_{722}$	0.49 **
	Dr	$D_{722}$	0.62 **
	Dr_min_	$D_{670}$	−0.60 **
	NDDr	$D_{722},D_{670}$	0.69 **
	RDr	$D_{722},D_{670}$	−0.67 **
	DDr	$D_{722},D_{670}$	0.55 **
	SDr	-	−0.65 **

Significance levels: ** 0.01.

**Table 4:** Estimation model of CNC based on the single spectral parameter.

Spectral Parameter	Regression Equations	Modeling Set		Verification Set	
		R^2^	RMSE	R^2^	RMSE
CI_rededge_	$y=2.4096e^{-0.113x}$	0.38	0.24	0.30	0.26
NDSI	$y=-625.3x^{3}$ $+498.6x^{2}-132.4x+14.4$	0.56	0.18	0.50	0.25
RSI	$y=-9.495x^{3}$ $+49.69x^{2}-86.77x+53.23$	0.55	0.17	0.54	0.18
DSI	$y=2.8882e^{-47.339x}$	0.40	0.20	0.35	0.20
NDDr	$y=0.253/\left(x^{2}-1.933x+1.028\right)$	0.52	0.19	0.53	0.18
RDr	y=−0.204lnx+1.6965	0.45	0.20	0.40	0.22
SDr	y=449x+2.646	0.44	0.25	0.43	0.30

**Table 5:** Estimation model of CNC based on the combination of multiple spectral parameters.

Types of Variable	PLSR		BPNN	
	R^2^	RMSE	R^2^	RMSE
Random two-band spectral indices	0.68	0.15	0.70	0.17
Red-edge parameters	0.54	0.17	0.66	0.17
Combination of random two-band spectral indices and red-edge parameters	0.64	0.16	0.77	0.16
